# Skin Treatment with Pulsed Monochromatic UVA1 355 Device and Computerized Morphometric Analysis of Histochemically Identified Langerhans Cells

**DOI:** 10.1155/2016/3415136

**Published:** 2016-07-20

**Authors:** Nicola Zerbinati, Federica Riva, Marco Paulli, Pier Camillo Parodi, Alberto Calligaro

**Affiliations:** ^1^Department of Surgical and Morphological Sciences, University of Insubria, 21100 Varese, Italy; ^2^Department of Public Health, Experimental and Forensic Medicine, Histology and Embryology Unit, University of Pavia, 27100 Pavia, Italy; ^3^Department of Molecular Medicine, University of Pavia and IRCCS San Matteo, 27100 Pavia, Italy; ^4^Department of Medical, Experimental and Clinical Sciences, University of Udine, 33100 Udine, Italy

## Abstract

Fluorescent or metal halide lamps are widely used in therapeutic applications in dermatological diseases, with broadband or narrow band emission UVA/UVA1 (320–400 nm) obtained with suitable passive filters. Recently, it has been possible for us to use a new machine provided with solid state source emitting pulsed monochromatic UVA1 355 nm. In order to evaluate the effects of this emission on immunocells of the skin, human skin samples were irradiated with monochromatic 355 nm UVA1 with different energetic fluences and after irradiation Langerhans cells were labeled with CD1a antibodies. The immunohistochemical identification of these cells permitted evaluating their modifications in terms of density into the skin. Obtained results are promising for therapeutical applications, also considering that a monochromatic radiation minimizes thermic load and DNA damage in the skin tissues.

## 1. Introduction

Langerhans cells are immunocompetent cells located in the epidermis of the skin. These cells have been provided with cytoplasmic processes intermingled with keratinocytes; they do not contain keratin and do not form desmosomes with keratinocytes. They represent a pool of cells which are part of the peripheral immunosystem playing a specific role in the pathogenesis of some inflammatory diseases of the skin including psoriasis [1, 2]. Langerhans cells are directly involved in the starting step of the complex mechanism of detection, processing of antigens contacting epidermis, and presentation of processed antigens to lymphocytes, with a crucial role as peripheral sentinels.

The use of nonionizing radiations, particularly in the UV range as elective treatment targeting Langerhans cells of many skin inflammatory diseases, has been extensively studied and applied in clinical practice [3].

Many devices using sources emitting wave lengths in the UV (A, B, and C), in the visible or infrared range targeting Langerhans cells, have been extensively used in clinical applications concerning atopic dermatitis, mycosis fungoides, scleroderma, lupus erythematosus, and psoriasis. However, the wavelengths and energetic fluences have to be tuned to the specific absorption characteristics of the irradiated tissues.

As different new devices have been recently introduced, we have studied and evaluated the effects of a pulsed monochromatic UVA1 treatment on Langerhans cells. These cells were identified with immunohistochemical method as CD1a-positive cells for CD1a (Cluster of Differentiation 1a antigen). Langerin is also a Langerhans cells marker but is expressed in mature cells. In histochemical preparations, the number of langerin-positive cells is lower than the number of CD1a-positive cells in the skin [4, 5], and therefore the evaluation of CD1a-positive cells was more suitable for morphometric evaluation of the UVA1 treatment effects on the eyelid skin here used.

## 2. Material and Methods

Eyelid samples from healthy subjects collected after surgical excision were irradiated (UVA1 355 nm) for 2 min, 3 min, and 4 min for a total transmitted energies, respectively, of 50, 75, and 100 J/cm^2^ and maintained with control samples 100% UVA1 protected (0 J/cm^2^) for 1 hour at 37°C in a culture medium optimized for skin [6].

For treatment, the energy UVA1 was produced by Alba 355 (EVLASER, Italy) based on a solid state laser (DPSS: Diode Pumped Solid State laser) used as an active medium and a coupled neodymium-doped yttrium orthovanadate (Nd:YVO4) crystal. The light emitted by the Nd:YVO4 at a wavelength of 1064 nm is sent to a crystal separator of 1064 nm harmonic components, particularly the second (532 nm) and the third (355 nm). By cutting off the second harmonic, the third harmonic emitted was filtered on. In this manner an emission of a single wavelength of 355 nm was obtained, as the UVA1 monochromatic wavelength used in this study.

After treatment, samples were fixed with a paraformaldehyde 4% solution PBS buffered, in order to obtain not only a good morphology but also the preservation of Langerhans cells specific antigens. Skin samples were then processed for microscopy through treatments of dehydration, paraffin embedding, microtome sectioning, rehydration, hematoxylin and eosin staining, last dehydration, and mounting on slides for observation. Particular care was used for a correct orientation of specimens in the embedding and sectioning procedures in order to have sections perfectly perpendicular to the skin surface.

Microscopic observations and digital recordings were made at a light microscope Carl Zeiss Axiophot provided with a 5-megapixel CCD camera Nikon DS-Fi2.

### 2.1. Immunohistochemistry

Antigenicity of the specific markers molecules, in particular CD1a (Cluster of Differentiation 1a), was well preserved with the fixative we have used, so immunohistochemistry with anti-CD1a antibody permitted the identification of Langerhans cells in the skin [7]. Tissue sections for immunohistochemistry were reacted with mouse monoclonal antibody anti-CD1a (DAKO, Agilent Technologies, USA). Slides were baked for 1 hour at 50°C, deparaffinized, and hydrated through a series of ethanol solutions at decreasing concentration to water. Slides were then treated with 0.05% Tween 20 in tris buffered saline for 5 minutes to permeabilize tissues and washed in tris buffered saline. Slides were heated in steamer in 1 mmol/L EDTA buffer (pH 8.0) or 10 mmol/L sodium citrate buffer (pH 6.0) for 20 minutes for antigen retrieval and treated with 3% hydrogen peroxide for 10 minutes to quench endogenous peroxidase activity. After washing with tris buffered saline, the slides were incubated with the primary antibody for 30 minutes at room temperature and then incubated with Envision1 (DAKO Corp.) anti-mouse labeled polymer conjugated with horseradish peroxidase for 20 minutes at room temperature. The immunolabeling reaction was revealed using DAB1 (Sigma-Aldrich, USA), and the sections were counterstained with hematoxylin. All immunohistochemical staining procedures were performed on an Autostainer Plus DAKO (DAKO Corp.).

### 2.2. Image Analysis

The selective immunocytochemistry staining not only permitted the identification of Langerhans cells but also permitted the application of a computerized morphometric analysis using ImageJ (NIH), a well-known software recognized as standard tool by international scientific community.

Computerized morphometric analysis was performed on immunostained sections with a thickness of 8 *μ*m, with a sampling area of 4 sqmm for each skin sample. Each section was digitally recorded at the microscope through a 5 Mpixels ccd camera at the same magnification and in the identical conditions of the color temperature of the light source. Resulting microphotographs were used for ImageJ morphometric analysis with the following steps:

(1) Equalization of the image extended to the level limits of 0 and 255 in order to detect and acquire, at the same extent for all slides, the finest structural stained details for the best selection of Langerhans cells in their whole extension.
(2) Segmentation of the continuous tone image is defining the most suitable threshold segmentation value for obtaining a binary image representing faithfully the Langerhans cells over a background of keratinocytes. For maintaining the full reliability of the specimens comparisons, the threshold values were the same for all slides.
(3) Measurements and collection of data and quantitative evaluation were performed. All results are presented in graph as mean percentage ± standard deviation in Figure 3. Statistical analysis was performed comparing samples with Student's *t*-test. Differences were considered significant at *p* < 0,01.

## 3. Results

Skin samples were UVA1 irradiated with energy fluences of 50, 75, and 100 J/cm^2^, and controls were 100% UVA1 protected (0 J/cm^2^). They were processed for histology and histochemistry all together in each step of the procedures, in order to minimize possible differences due to technical reasons and to assure a reliable comparative analysis.

**Figure 1:**
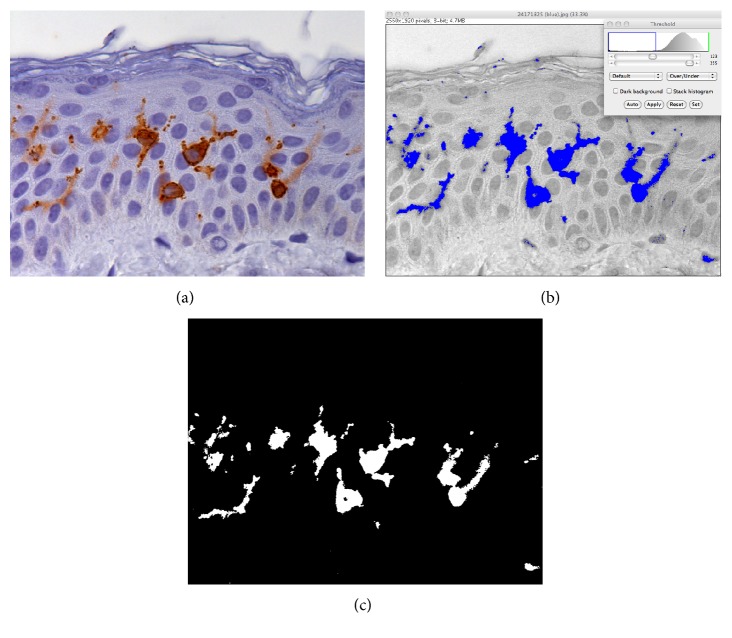
More relevant steps of the image analysis procedure illustrated on a skin control sample. (a) Immunohistochemical analysis identified CD1a-positive Langerhans cells (brown) on a background of keratinocytes faintly hematoxylin stained. (b) Continuous tone image with labeled CD1a-positive Langerhans cells, blue masked following segmentation (inset), ready for digital conversion. (c) Binary image resulting from digitalization where white areas are constituting the pool of pixels to be evaluated.

**Figure 2:**
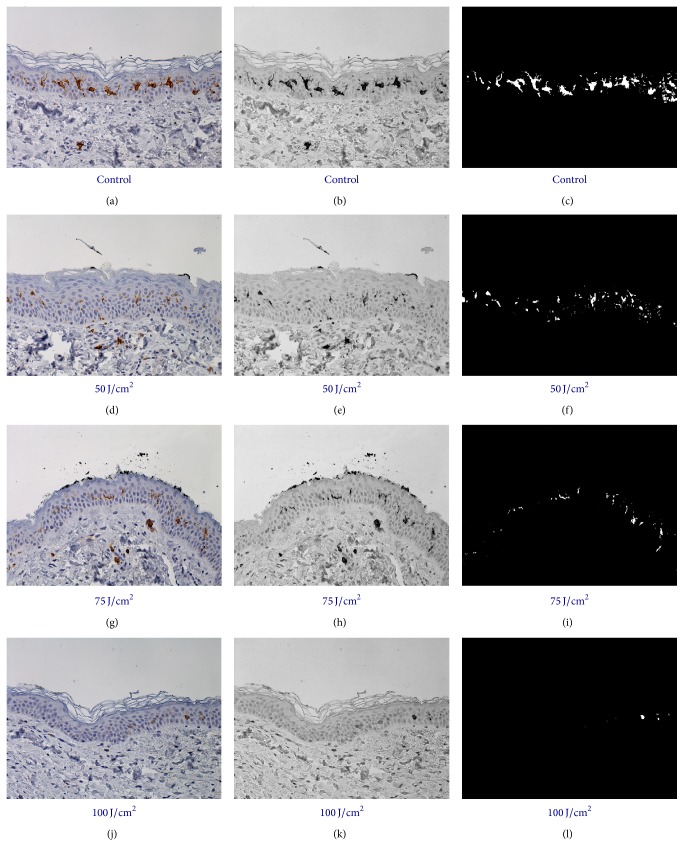
The same procedure of Figure 1 was applied to all samples: control (a, b, and c), and samples irradiated with fluences of 50, 75, and 100 J/cm^2^, respectively (d–l). Columns identify original images (a, d, g, and j), segmented images (b, e, h, and k), and digital images (c, f, i, and l).

In Figure 1, immunostaining and image analysis of a control section are reported. In Figure 1(a), Langerhans cells, body, and cytoplasmic processes, labeled by specific antibody for CD1a, appear clearly visible (brown) on the background of keratinocytes faintly stained with hematoxylin.  Figure 1(b) is representing the segmentation of the continuous tone image through a passband digital filter with related blue mask.  Figure 1(c) shows the digitalized image where white pixels are constituting the areas to be measured as percentage of the 2560 × 1920 pixels standard reference frame. This methodological way has been applied with the same segmentation parameters to all sections obtained from controls (Figures 2(a), 2(b), and 2(c),  100% protected) and from specimens irradiated with energy fluences of 50, 75, and 100 J/cm^2^, respectively (Figures 2(d)–2(l)). Finally (Figures 2(c), 2(f), 2(i), and 2(l)), the white areas of the digital images representing CD1a-positive Langerhans cells were measured, as percentage of the full area of the same reference frame.

**Figure 3:**
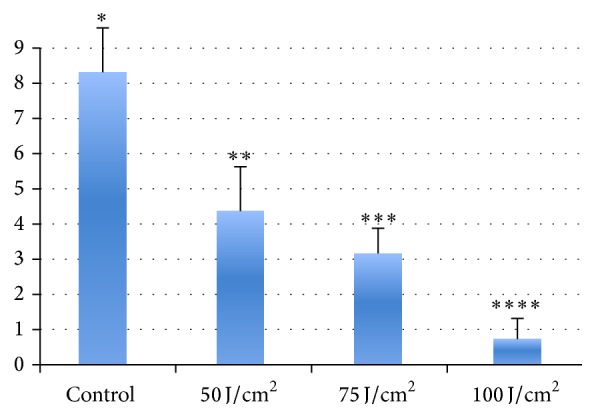
Percentage of CD1a-positive Langerhans cells in skin control and treated with monochromatic UVA1 355. Results of the measurements, where the units represent the percentage areas occupied by CD1a-positive Langerhans cells in controls and irradiated, respectively, over the same reference areas. Student's *t*-test results: *∗* versus *∗∗*, *∗∗* versus *∗∗∗*, and *∗∗∗* versus *∗∗∗∗*, for all *p* < 0,01.

The results of the measurements are reported as Figure 3, where the immunoreactivity for CD1a, covering 8,34%  (SD ± 1,12) of the frame in controls, appears to be reduced to 4,37%  (SD ± 1,24) at 50 J/cm^2^, to 3,18%  (SD ± 0,86) at 75 J/cm^2^, and to 0,73%  (SD ± 0,48) at 100 J/cm^2^. Statistical analysis between samples showed significative differences (*p* < 0,01, asterisks in Figure 3). The reduction of the CD1a labeled Langerhans cells areas is related to the energetic fluence, with a maximal reduction after treatment with energetic fluence of 100 J/cm^2^.

## 4. Discussion and Conclusions

The fluorescent lamps have low irradiance (5–10 W/cm^2^) and delivery low UVA dose in long time (10–30 J/cm^2^). High-output metal halide source has a much greater irradiance (80 W/cm^2^) and can irradiate medium or high doses in treatment sessions of 50 min. Conventional machines for UVA treatment emit wavelengths mainly between 340 and 400 nm but may also produce scattered radiation >530 nm including infrared irradiation (780–3000 nm). Conventional UVA phototherapy may be accompanied by extensive heat load predominantly generated by infrared irradiation (780–3000 nm) and/or insufficient cooling systems of the phototherapy devices.

Light therapy lamps with halogen-metal band confined to the very high irradiance UVA1 (340–400 nm) have experienced a growing success and interest [8]. Unlike UVA2 and UVB (290–340 nm), the biological effects of UVA1 on the skin are mainly related to oxidative stress, mediated by aerobic photooxidation with the intermediate formation of reactive oxygen species such as singlet oxygen, superoxide anion, and hydroxyl radicals [9–11].

About 37% of Caucasian UVA1 irradiation penetrates deeply in the skin up to 60–90 mm and therefore these wavelengths can effectively modulate the activity of immune and inflammatory cell populations resident or patrolling the epidermis and the dermis.

UVA1 may induce apoptosis of T lymphocytes, especially T helper, not only through late mechanisms dependent on a synthesis* de novo* of some proteins but also through mechanisms that are early, preprogrammed, and independent of the UVA1 proteins synthesis. This is causing the production of PGE2 and thromboxane in the cells of Langerhans but does not alter the number and function and induces migration in satellite lymph nodes [3].

The recent availability of a machine emitting pulsed monochromatic UVA1 355 nm wavelength (Alba 355) allowed evaluating the effects of such irradiation on CD1a-positive Langerhans cells, considering that a monochromatic radiation minimizes collateral effects in the irradiated tissues. The radiation used (not a broad or narrow band but a monochromatic wave) and the very short time of single pulses permitted a much better control of the energy transfer to the skin. In this way, at the same time, it minimizes thermal surcharge of tissues and risks related to DNA damage, with pyrimidine dimers formation [12]. Our experimental model has been eyelid skin fragments freshly excised and maintained in short term organotypic culture (one hour, comprehensive of the short time of irradiation). The protocols for UVA1 355 nm phototherapy of skin samples involved the irradiation of single specimens with 50, 75, and 100 J/cm^2^, respectively, and specimens 100% protected as control.

As the most common and reliable antigen for the histochemical identification of Langerhans cells in the epidermis, we have considered the CD1a antigen, a transmembrane glycoprotein structurally related to the Major Histocompatibility Complex (MHC) protein antibody currently used as specific marker of these cells [13]. CD1a, also expressed on macrophages on the connective tissue, is highly specific for Langerhans cells in the epidermis and used in routine dermatological diagnostic practice. Our* in vitro* experimental condition of the skin samples and the short time following irradiation did not permit considering the possible migration of these cells in the dermis and towards lymph nodes.

Our results are demonstrating significant modifications of the percentage of anti-CD1a labeled cells in the epithelium of skin treated with specific 355 nm monochromatic wavelength, with an effect energy-transfer dependent, minimizing collateral adverse effects on DNA differently from wide spread UVA applications in dermatological diseases [11].
